# Seed filling under different temperatures improves the seed vigor of hybrid rice (*Oryza sativa* L.) via starch accumulation and structure

**DOI:** 10.1038/s41598-020-57518-5

**Published:** 2020-01-17

**Authors:** Xiaomin Wang, Huabin Zheng, Qiyuan Tang, Qimin Chen, Wenwei Mo

**Affiliations:** 1 College of Agronomy, Hunan Agricultural University, Changsha, 410128 China; 2 Yibin Vocational and Technical College, Sichuan, 644000 China

**Keywords:** Seed development, Seed distribution, Seed development, Seed distribution

## Abstract

Seed filling is crucial for seed vigor and starch accumulation and structure. Differences in hybrid rice seed vigor were evaluated in field experiments, conducted across two sites in 2017 and 2018, under different seed filling temperatures along with the underlying mechanisms related to the seed filling characteristics and starch accumulation and structure. Significant differences in the seed vigor parameters were revealed, with different seed filling characteristics observed under different temperatures. When averaged across cultivars, the seeds with a low seed filling rate and long seed filling duration obsessed 11.9% higher germination percentage (GP) and 22.7% higher vigor index (VI) than those with a high seed filling rate and short seed filling duration. Moreover, a high seed filling rate and short seed filling duration significantly decreased the total starch and amylose contents and increased the amylopectin content. Additionally, when averaged across cultivars, the relative crystallinity and starch granule diameter obtained with a high seed filling rate and short seed filling duration were 3.8% and 15.1% higher, respectively, than those with a low seed filling rate and long seed filling duration. In summary, it can be speculated that seed filling characteristics determine hybrid rice seed vigor by affecting starch accumulation and structure.

## Introduction

Rice (*Oryza sativa* L.) is one of the most important crops in China. With increasing human populations and decreasing areas of agricultural land, the promotion of rice production, especially hybrid rice production, is essential in China^1^. Seed vigor, which refers to the potential of seed to germinate rapidly and uniformly under a wide range of field conditions, is an essential requirement for agricultural production^2,3^. Vigorous seeds of hybrid rice, which are characterized by a fast germination, high gemination percentage and uniform seedling growth, are beneficial for increasing grain yields and profits for farmers^2^. In contrast, the hybrid rice seeds with low seed vigor would result in poor crop establishment and lead to great yield losses, suggesting that the seed vigor plays a vital role in the realization of hybrid rice productivity.

The seed vigor of hybrid rice can be determined by several factors including seed filling, which is characterized by the duration and rate of seed filling^4^. The filling speed of hybrid rice seeds is initially slow, then a rapid filling phase occurs, and then growth slows again with maturity^5^. Previous studies have reported that the grain growth of hybrid rice is associated with certain grain filling characteristics^6,7^. A high seed filling rate and short seed filling duration limit grain growth and development and contribute to the losses of grain weight and seed yield^7–10^. Moreover, Abayawickrama *et al*.^11^ and Liu *et al*.^12^ found that grain quality was affected by grain filling characteristics formed under different temperatures. Therefore, seed filling influences seed growth and development and ultimately affects the seed quality of hybrid rice. Nevertheless, the variation in seed vigor with different seed filling characteristics has rarely been explored by previous studies.

Starch is predominantly present in the endosperm cells of mature rice seeds^13,14^. Additionally, starch is deposited in seeds in the form of granules, which are approximately 3–8 μm each and angular in shape in rice seeds^15^. Moreover, relevant evidence indicates that rice starch formation is influenced by grain development^16,17^. Shu *et al*.^16^ found that grain filling affected the formation of resistant starch by comparing three rice mutants with different contents of resistant starch. Cai *et al*.^17^ reported that different morphologies of rice starch were observed during grain filling period and ultimately affected grain quality. However, knowledge is limited about the effects of seed filling on starch accumulation and structure during hybrid rice seed production.

We hypothesized that seed filling characteristics contributed to the variations in seed vigor of hybrid rice by affecting starch accumulation and structure. Therefore, the objectives of this study were to evaluate the differences in seed vigor of hybrid rice under different temperatures during seed filling and to explore the mechanisms associated with such variations with regard to seed filling characteristics and starch accumulation and structure.

## Results

### Daily mean temperature, daily minimum temperature and daily maximum temperature

**Figure 1:**
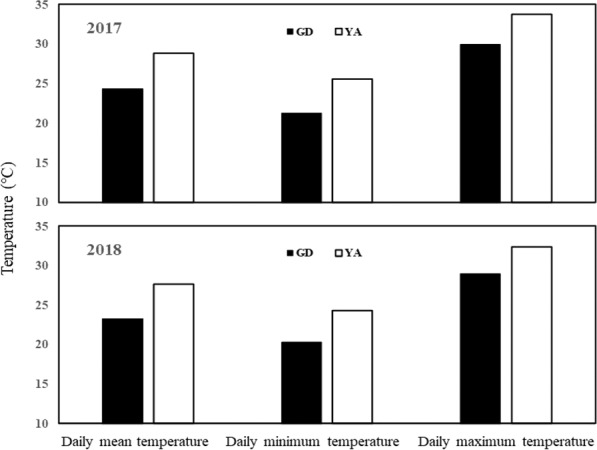
Daily mean temperature, daily minimum temperature and daily maximum temperature during seed filling in 2017 and 2018. GD and YA indicate Guidong and Yongan, respectively.

The daily mean temperature, daily minimum temperature and daily maximum temperature in Yongan (YA) were markedly higher than those in Guidong (GD) during hybrid rice seed filling period (Fig. 1). The variation in temperature remained relatively constant in both years. The daily mean temperature in GD was 24.4 °C and 23.5 °C in 2017 and 2018, respectively. The daily mean temperature in YA was 28.8 °C and 27.8 °C in 2017 and 2018, respectively. In 2018, the values for daily mean temperature, daily minimum temperature, and daily maximum temperature in YA were 18.3%, 19.7%, and 11.7% higher than GD, respectively. Therefore, higher temperatures were recorded in YA during hybrid rice seed filling period.

### Seed weight, seed filling duration and seed filling rate

Significant differences in the seed filling duration and seed filling rate were observed, while a non-significant difference was obtained for the final seed weight under the different temperatures (Table 1). The final seed weight in GD was higher than YA for both varieties. Moreover, the seed filling duration in GD was 32.2% higher than YA for IIY838 and 35.1% higher for IIY416. However, the seed filling rate in GD was 25.0% lower than YA for IIY838 and 21.4% lower for IIY416. The determination coefficients (R^2^) for the seed filling process ranged from 0.9677 to 0.9863.

**Table 1:** Final seed weight, seed filling duration and seed filling rate in 2018.

Variety	Site	Final seed weight (mg grain^−1^)	Seed filling duration (day)	Seed filling rate (mg grain^−1^ day^−1^)	R2	P Value
IIY838	GD	24.0a	23.0a	0.9b	0.9833	<0.01
	YA	23.6a	17.4b	1.2a	0.9677	<0.01
IIY416	GD	24.7a	20.8a	1.1b	0.9861	<0.01
	YA	23.8a	15.4b	1.4a	0.9863	<0.01

Data are mean values of three replications. Different lowercase letters denote significant differences at different sites of the same variety at the 0.05 probability level according to the LSD test. R^2^ indicates the adjusted coefficient of determination. IIY838 and IIY416 indicate IIyou838 and IIyou416, respectively. GD and YA indicate Guidong and Yongan, respectively.

### Germination percentage (GP) and vigor index (VI)

Significant differences in the GP and VI were obtained at different sites (Table 2). The GP and VI in GD were considerably higher than YA for IIY838 and IIY416 in both years. The mean GP in GD was 15.5% higher than YA for IIY838 and 8.2% higher for IIY416. Moreover, the mean VI in GD was 25.9% higher than YA for IIY838 and 19.4% higher for IIY416.

**Table 2:** Germination percentage (GP) and vigor index (VI) in standard germination test.

Year	Variety	Site	GP (%)	VI
2017	IIY838	GD	88.7 ± 1.8	10.4 ± 0.2
		YA	73.3 ± 1.9	8.4 ± 0.3
	IIY416	GD	89.0 ± 2.0	11.2 ± 0.2
		YA	82.7 ± 1.5	9.7 ± 0.3
2018	IIY838	GD	88.3 ± 0.9	10.1 ± 0.3
		YA	72.7 ± 1.7	7.7 ± 0.5
	IIY416	GD	87.3 ± 0.3	10.9 ± 0.1
		YA	77.3 ± 1.3	8.9 ± 0.2
Mean	IIY838	GD	88.5 ± 0.2a	10.2 ± 0.2a
		YA	73.0 ± 0.3b	8.1 ± 0.3b
	IIY416	GD	88.2 ± 0.8a	11.1 ± 0.2a
		YA	80.0 ± 2.7b	9.3 ± 0.4b

Data are mean values ± SE (n = 4). Different lowercase letters denote significant differences at different sites of the same variety at the 0.05 probability level according to the LSD test. IIY838 and IIY416 indicate IIyou838 and IIyou416, respectively. GD and YA indicate Guidong and Yongan, respectively.

### Total starch content, amylose content and amylopectin content

**Figure 2:**
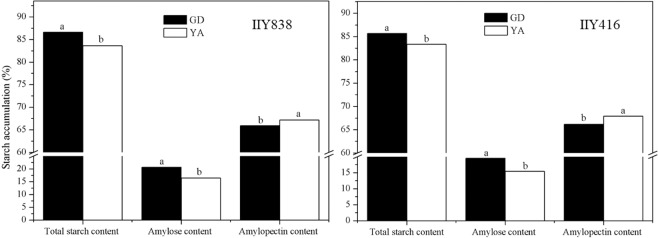
Total starch content, amylose content and amylopectin content of hybrid rice varieties in 2018. Different lowercase letters denote significant differences at different sites of the same variety at the 0.05 probability level according to the LSD test. GD and YA indicate Guidong and Yongan, respectively.

A significant difference in starch accumulation was obtained with different seed filling characteristics (Fig. 2). The values for total starch content and amylose content in GD were significantly higher than YA, whereas the value for amylopectin content in GD was significantly lower than YA. Moreover, the value for amylose content in GD was 4.3% higher than YA for IIY838 and 4.1% higher for IIY416. The value for total starch content in GD was 3.0% higher than YA for IIY838 and 2.4% higher for IIY416.

### Starch granule morphology and granule size distribution

**Figure 3:**
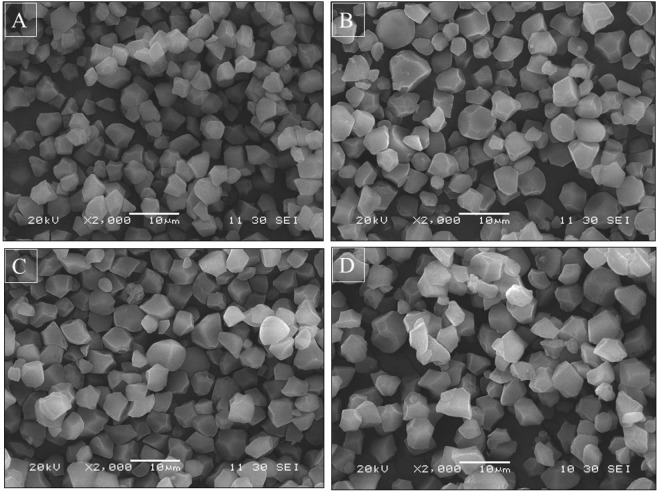
Morphology of hybrid rice seed starch in GD and YA. (**A**) Starches of IIY838 in GD; (**B**): Starches of IIY838 in YA; (**C**): Starches of IIY416 in GD; (**D**): Starches of IIY416 in YA. IIY838 and IIY416 indicate IIyou838 and IIyou416, respectively. GD and YA indicate Guidong and Yongan, respectively.

Isolated starch samples were observed using a scanning electron microscope, and the starch granules of the hybrid rice seed were polygonal in GD and YA (Fig. 3). Starch granules of both varieties from the different sites had a smooth surface and were pleomorphic. However, starch granules of IIY838 in GD (Fig. 3A) were smaller than those in YA (Fig. 3B). The starch granules of IIY416 in GD (Fig. 3C) were more tightly packed and compact and exhibited fewer spaces between each other, while those in YA (Fig. 3D) were round with large spaces between each other.

Significant variations in the particle diameter of hybrid rice seed starches were observed with the different seed filling characteristics (Table 3). The values for diameter of starch granules in GD were smaller than YA for both varieties. The D (3, 2) value in YA was 15.4% higher than GD for IIY838 and 16.2% higher for IIY416. The D (4, 3) value in YA was 15.6% higher than GD for IIY838 and 14.4% higher for IIY416. The D (50) value in YA was 14.3% higher than GD for IIY838 and 15.8% higher for IIY416.

**Table 3:** Diameter of starch granule at different sites in 2018.

Variety	Site	D (3, 2)	D (4, 3)	D (50)
IIY838	GD	4.55 ± 0.06b	4.82 ± 0.06b	4.77 ± 0.05b
	YA	5.25 ± 0.05a	5.57 ± 0.07a	5.45 ± 0.09a
IIY416	GD	4.58 ± 0.08b	4.93 ± 0.04b	4.82 ± 0.04b
	YA	5.32 ± 0.06a	5.64 ± 0.06a	5.58 ± 0.07a

Data are mean values ± SE (n = 3). Different lowercase letters denote significant differences at different sites of the same variety at the 0.05 probability level according to the LSD test. D (3, 2) is the surface-area weighted mean diameter. D (4, 3) is the volume-weighted mean diameter. D (50) is the average particle size of the starch samples. IIY838 and IIY416 indicate IIyou838 and IIyou416, respectively. GD and YA indicate Guidong and Yongan, respectively.

### X-ray diffraction patterns of hybrid rice seed starches

**Figure 4:**
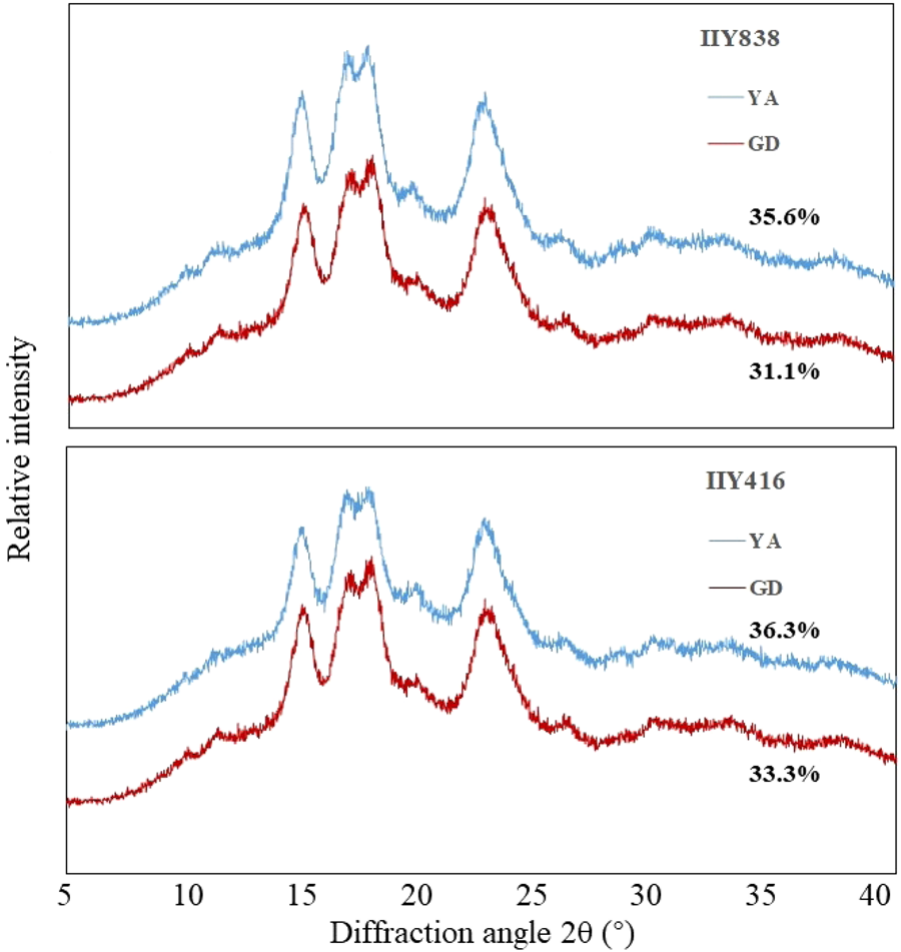
X-ray diffraction patterns of hybrid rice seed starch in GD and YA. IIY838 and IIY416 indicate IIyou838 and IIyou416, respectively. GD and YA indicate Guidong and Yongan, respectively.

Starches of the hybrid rice seeds showed the same A-type crystalline structure with the major X-ray diffraction peaks at 15°, a doublet at 17° and 18°, and 23° (2θ) (Fig. 4). However, the relative crystallinity of starches in YA was higher than that in GD for both varieties. The relative crystallinity in YA was 4.5% higher than GD for IIY838 and 3.0% higher for IIY416.

## Discussion

It is widely known that grain filling characteristics are associated with the temperature during grain growth and development^11,18,19^. In this study, the temperature during seed filling period in Yongan (YA) was remarkably higher than that in Guidong (GD) (Fig. 1). The considerable variation in the temperature resulted in different seed filling characteristics, irrespective of the hybrid rice varieties. A low seed filling rate and long seed filling duration occurred in GD, whereas a high seed filling rate and short filling duration occurred in YA (Table 1). These results indicated that high temperatures accelerated the rate of seed filling and shortened the duration of seed filling during hybrid rice seed production. Many corroborating results have been reported in previous studies^4,20–23^. Moreover, high temperatures during grain filling will adversely cause stress on seed growth and development^24,25^. In this study, the final seed weight in GD was higher than YA for both varieties (Table 1). Tashiro and Wardlaw^26^ reported that the grain weight of *Japonica* rice cultivars was obviously reduced when the daily mean temperature exceeded 26 °C during the grain filling period. Kato *et al*.^14^ found that seed weight was significantly decreased at high ripening temperatures (a daily mean temperature of 28 °C). Additionally, previous studies have reported that the decrease in grain weight due to high temperature has been ascribed to the shortening of the grain filling duration^20,21,27^. Therefore, the seed filling characteristics observed in GD were more suitable for seed growth and development than those in YA.

The period from seed filling to maturity is critical for the development of seed vigor during hybrid rice seed production. In this study, the seed vigor parameters, including the germination percentage (GP) and vigor index (VI), in GD were significantly higher than YA for both varieties (Table 2). Ito *et al*.^28^ and Chen *et al*.^29^ reported that the high temperature at the grain filling period will decrease the quality of the rice. Moreover, a high seed filling rate and short seed filling duration cause stress, impacting seed growth and development^8,9^. Therefore, it can be speculated that a lower seed filling rate and longer seed filling duration observed at low temperature during seed filling period could positively increase the seed vigor of hybrid rice during seed production.

Starch is the main compound in rice seeds, and seed filling is critical to starch formation and quality^19,30,31^. In this study, the total starch content and amylose content in GD were significantly higher than YA, whereas the amylopectin content in GD was significantly lower than YA (Fig. 2). Tang *et al*.^32^ found that temperatures during grain development had a significant effect on the accumulation of amylose and amylopectin. Kato *et al*.^14^. reported that the higher temperature decreased the apparent amylose content and increased the ratio of short to long chains of amylopectin. These results indicated that a low seed filling rate and long seed filling duration were beneficial to starch accumulation, especially for the total starch content and amylose content. Moreover, it has reported that the amylose content is positively related to seed vigor in hybrid rice seeds^33^. Thus, the high amylose content in seeds was responsible for the higher GP and VI in GD. In this study, the relative crystallinity of starches in YA was higher than GD in both hybrid rice seeds (Fig. 4). The diameter of starch granules in GD was smaller than YA for both varieties (Table 3). Liu *et al*.^12^ found that the average diameter of starch granules at high temperature was significantly higher than those at lower temperature during grain filling period. Additionally, many studies have indicated that higher temperatures mainly affect changes in starch morphology and structure, and will directly affect the final rice quality^34^. Therefore, different seed filling characteristics would change the seed starch structure and ultimately affect seed vigor during hybrid rice seed production.

In this study, we utilized the Richards equation to characterize the seed filling process during hybrid seed production. The results showed a high value of R^2^ (0.9677–0.9863), indicating that the equation could be used to describe the seed filling process of hybrid rice seeds. This was in agreement with previous reports that modelled the grain filling process with the Richards’s equation^16,35,36^.

## Conclusions

During hybrid rice seed production, the seeds with a low seed filling rate and long seed filling duration obsessed the higher germination percentage (GP) and vigor index (VI) than those with a high seed filling rate and short seed filling duration. Moreover, significant differences in total starch content, amylose content, amylopectin content, relative intensity and diameter of starch granules were obtained, with different seed filling characteristics observed under different temperatures. Therefore, it can be speculated that different seed filling characteristics result in changes in starch accumulation and structure and ultimately influence seed vigor.

## Materials and Methods

### Experimental sites and hybrid combinations

Field experiments were conducted in 2017 and 2018 at Yongan (YA; 28°09′N, 113°37′E) and Guidong (GD; 25°08′N, 113°91′E), respectively, on research farms of Hunan Agricultural University, Hunan Province, China. The soil at the site of the YA 2018 experiment was a tidal clay (Fluvisol, FAO taxonomy) with the following properties: pH = 5.75, organic matter = 34.4 g kg^−1^, available N = 81.8 mg kg^−1^, available P = 34.4 mg kg^−1^, and available K = 56.2 mg kg^−1^. The soil at the site of the GD 2018 experiment was a tidal clay (Fluvisol, FAO taxonomy) with the following properties: pH = 5.35, organic matter = 48.8 g kg^−1^, available N = 135.7 mg kg^−1^, available P = 43.6 mg kg^−1^, and available K = 74.3 mg kg^−1^.

The experimental hybrid combinations used in this study were IIyou838 (II-32A as the female parent × R838 as the male parent; IIY838) and IIyou416 (II-32A as the female parent × R416 as the male parent; IIY416). IIY838 and IIY416 were obtained from Longping Seed Industry Co., Ltd. in Hunan Province, China. The climate of the parent seed production sites was moist subtropical monsoon.

## Experimentation

### Field experiments

Plots were laid out in a randomized complete block configuration with three replications, and each plot was 6 m × 10 m in size. Ten uniform hills from each plot were harvested at anthesis, and 5, 10, 15, 20, 25, 30 and 35 days after anthesis. Panicles separated from the plants were hand-threshed into rachises and spikelets. Filled spikelets were separated from unfilled spikelets by submersion in tap water and were oven-dried at 70 °C to a constant weight for the determination of the dry weight^37^. At the final harvest, ten additional hills from each plot were harvested and hand-threshed into rachises and spikelets. Filled spikelets separated from unfilled spikelets were dried to a moisture content of 13% with sunshine. Dried filled spikelets stored in mesh bags at room temperature for three months. Then, the total starch content, amylose content, amylopectin content and starch structure were measured. Data of daily mean temperature, daily minimum temperature and daily maximum temperature were recorded from the weather station located 100 m from the experimental field.

Parental seedlings were raised on nursery beds and transplanted manually. The sowing and transplanting date are presented in Table 4, and the male parents were sowed twice. The row and plant spacing of the female parents was 133 mm × 200 mm, and 3–4 seeds were sown per plant. The row and plant spacing of the male parent was 167 mm × 300 mm, and 2–3 seeds were sown per plant. The parental row ratio was 2:12, and the spacing of the parent was 333 mm. Gibberellic acid (GA_3_) was applied (300 g hm^−2^) to both varieties at anthesis, which is generally done, to change the structure of hybrid combination populations in hybrid rice seed production. The GA_3_ applied in the field experiments was purchased from Longping Seed Industry Co., Ltd. in Hunan Province, China. Fertilizer, pesticide and herbicide management followed local practices for hybrid rice seed production.

**Table 4:** Sowing and transplanting date of varieties in 2017 and 2018.

Year	Site	Variety	Female/Male	Sowing date	Transplanting date
2017	GD	IIY838	II-32A	05-06	06-01
			R838	05-08	06-01
				05-12	06-01
		IIY416	II-32A	05-06	06-01
			R416	04-29	05-29
				05-04	05-29
	YA	IIY838	II-32A	05-11	06-02
			R838	05-09	06-02
				05-15	06-02
		IIY416	II-32A	05-11	06-02
			R416	05-04	05-30
				05-09	05-30
2018	GD	IIY838	II-32A	05-06	06-01
			R838	05-08	06-01
				05-12	06-01
		IIY416	II-32A	05-06	06-01
			R416	04-29	05-29
				05-04	05-29
	YA	IIY838	II-32A	05-11	06-02
			R838	05-09	06-02
				05-15	06-02
		IIY416	II-32A	05-11	06-02
			R416	05-04	05-30
				05-09	05-30

IIY838 and IIY416 indicate IIyou838 and IIyou416, respectively. GD and YA indicate Guidong and Yongan, respectively.

### A seed germination test

The seed germination test was conducted after three months from harvest, and seed vigor parameters were measured according to the method described by Wang *et al*.^38^ and ISTA^39^ with minor modifications. A number of 100 healthy seeds from each sample with four replications were soaked in 0.6% (6 g/L) sodium hypochlorite solution for 15 min in order to sterilize the seeds. The disinfectant seeds were then rinsed with sterile distilled water. The seeds were placed on two layers of filter papers in a plastic box (12 cm × 12 cm × 5 cm). Then 9 ml of distilled water was added in the plastic box. The plastic boxes were placed in a growth chamber with 12-/12-h light/dark cycles at the temperature of 30 °C for 7 days. Normally germinated seeds were recognized when the root length reached the seed length and the shoot length reached half of the seed length^38^. The number of germinated seeds was counted daily for 7 days. The sum of the daily counts was defined as the germination percentage (GP). The vigor index (VI) was calculated using the equation:

1 $$VI=DW\times \sum (Gt/t)$$

where *DW* represents the dry weight of the seedlings of germinated seeds and *Gt* represents the number of germinated seeds on day *t*^40^.

### Observations and measurements

#### Starch isolation

Starch of rice seeds was isolated referring to the method described by Wei *et al*.^41^ with some modifications. Firstly, a solution was prepared that 10 mg g^−1^ of alkaline protease was added in the 0.45% aqueous solution of sodium metabisulfite. Then 10 g of the sample was soaked in the prepared solution for 24 h in order to remove the protein in the rice flour. The starch slurry was filtered through a 200-mesh sieve. The residue collected from the mesh sieve was mixed with 30 mL of deionized water and stirred for 2 min. Then the mixture was filtered through a 200-mesh sieve. Starch slurry filtrates was combined and then centrifuged at 3000 g for 10 min. The faint-colored supernatant in the starch slurry was then discarded after centrifugation. Before the supernatant was again removed, the precipitate was mixed with 20 mL of deionized water and centrifuged at 3000 g for 10 min. In order to thoroughly remove impurities, the centrifugation process was repeated five times. Afterwards, the starch of rice seeds was dried at 30 °C under ambient pressure. The dried starch of rice seeds then sieved (200 mesh).

#### Regression analyses for the seed filling rate and seed filling duration

The seed filling process of rice seeds was estimated by the Richards^42^ growth equation, as previously described by Yang *et al*.^35^, to calculate seed filling rate and seed filling duration as a function of degree days:

2 $$W=\frac{A}{{(1+B{e}^{-kt})}^{1/N}}$$

where *W* represents the seed weight, *t* represents the days after anthesis, *A* represents the final seed weight and *B*, *k*, and *N* are constants and determined by the coefficients of the regression. The seed filling duration was defined as the period when *W* was from 5% (*t*_1_) to 95% (*t*_2_) of *A*. The average seed filling rate during the period was calculated from *t*_1_ to *t*_2_.

#### Measurements of total starch content, amylose content and amylopectin content

The percent of the total starch was determined by a spectrophotometric method^43^. The percent of amylose was measured according to the simplified amylose assay procedure of Juliano^44^ and Raja *et al*.^45^ using the spectrophotometric method. The amylopectin content was equal to the total starch content minus the amylose content.

#### Observation of starch granules microstructural characteristics

The microstructural characteristics of starch granules were observed by scanning electron microscopy (SEM JSM-6380LV, Joel Ltd., Tokyo, Japan), and according to the method described by Lu *et al*.^19^ with minor modifications. Starch samples were fixed on a conductive adhesive and coated with gold using a sputter coater. Then starch samples were observed and photographed by scanning electron microscopy at an accelerating voltage of 20 kV.

#### Measurements of starch particle size

The starch particle size was obtained with a laser particle size analyzer (LS-POP6, OMEC Instruments Co. Ltd., Guangzhou, China), and according to the method described by Liu *et al*.^12^ with minor modifications. The refractive index of starch samples was set at 1.6. The refractive index of water was set at 1.33. The average particle size of the starch samples was recorded as D (50). The surface-area weighted mean diameter of the starch samples was recorded as D (3,2). The volume-weighted mean diameter of the starch samples was recorded as D (4,3).

#### X-ray diffraction analysis

The X-ray patterns of rice seed starches were measured by an X-ray diffractometer (XRD-6100, Shimadzu Co. Ltd., Japan), and according to the method described by Wei *et al*.^41^ with minor modifications. The starch powder was packed tightly into small holders. The scanning range was from 5° to 40° with 2θ values. The scanning speed was at the rate of 2.0°/min. The software MDI Jade 6 was used to calculate the relative crystallinity. The different peaks of starch X-ray diffraction patterns and the peaks into the corresponding areas were analyzed and fitted, respectively, using the software MDI Jade 6. The ration of the peak area to the sum of the peak area and the background area was defined as the relative crystallinity.

### Data analysis

Data were analyzed using the analysis of variance (ANOVA) procedure in Statistix 8.0 (analytical software, Tallahassee, FL, USA), and multiple comparisons were explored using Fisher’s protected least-significant difference (LSD) test at the 0.05 probability level. Before analysis, the percentage data were arcsine-transformed. In regression analyses, the adjusted coefficient of determination R^2^ was used.

## References

[1] Ma GH, Yuan LP (2015). Hybrid rice achievements, development and prospect in China. J. Integr. Agr..

[2] Sun Q, Wang JH, Sun BQ (2007). Advances on seed vigor physiological and genetic mechanisms. J. Integr. Agr..

[3] Finch-savage WE, Bassel GW (2015). Seed vigour and crop establishment: extending performance beyond adaptation. J. Exp. Bot..

[4] Yang WH, Peng SB, Maribel LDS, Rebecca CL, Romeo MV (2008). Grain filling duration, a crucial determinant of genotypic variation of grain yield in field-grown tropical irrigated rice. Field Crop. Res..

[5] Yoshida, S. *Fundamentals of Rice Crop Science*. International Rice Research Institute, Los Banos, Philippines, 59–60 (1981).

[6] Wang Y (2017). Characteristics of starch synthesis and grain filling of common buckwheat. J. Cereal Sci..

[7] Masaki O (2018). Characterization of high-yielding rice cultivars with different grain-filling properties to clarify limiting factors for improving grain yield. Field Crop. Res..

[8] Kobata T, Yoshida H, Mssiko U, Honda T (2013). Spikelet sterility is associated with a lack of assimilate in high-spikelet-number rice. Agron. J..

[9] You CC (2016). Effect of removing superior spikelets on grain filling of inferior spikelets in rice. Front. Plant Sci..

[10] Yoshinaga S, Takai T, Arai-Sanoh Y, Ishimaru T, Kondo M (2013). Varietal differences in sink production and grain-filling ability in recently developed high-yielding rice (*Oryza sativa* L.) varieties in Japan. Field Crop. Res..

[11] Abayawickrama ASMT, Reinke RF, Fitzgerald MA, Harper JDI, Burrows GE (2017). Influence of high daytime temperature during the grain filling stage on fissure formation in rice. J. Cereal Sci..

[12] Liu JC (2017). Influence of environmental temperature during grain filling period on granule size distribution of rice starch and its relation to gelatinization properties. J. Cereal Sci..

[13] Tester RF, Karkalas J, Qi X (2004). Starch–composition, fine structure and architecture. J. Cereal Sci..

[14] Kato K (2019). Effect of high temperature on starch biosynthetic enzymes and starch structure in japonica rice cultivar ‘Akitakomachi’ (*Oryza sativa* L.) endosperm and palatability of cooked rice. J. Cereal Sci..

[15] Luca A, Jonathan OR, Alan LK, James AO (2016). Chemistry, structure, functionality and applications of rice starch. J. Cereal Sci..

[16] Shu XL, Sun J, Wu DX (2014). Effects of grain development on formation of resistant starch in rice. Food Chem..

[17] Cai Y, Liu C, Wang W, Cai K (2011). Differences in physicochemical properties of kernels of two rice cultivars during grain formation. J. Sci. Food Agr..

[18] Lu DL (2013). Effects of high temperature during grain filling under control conditions on the physicochemical properties of waxy maize flour. Carbohydr. Polym..

[19] Lu DL, Yang H, Shen X, Lu WP (2016). Effects of high temperature during grain filling on physicochemical properties of waxy maize starch. J. Integr. Agr..

[20] Nagato K, Ebata M (1965). Effect of high temperature during ripening period on the development and the quality of rice kernels. Japanese J. Crop Sci..

[21] Tashiro T, Wardlaw I (1989). A comparison of the effect of high temperature on grain development inn wheat and rice. Ann. Bot..

[22] Kim J (2011). Relationship between grain filling duration and leaf senescence of temperate rice under high temperature. Field Crop. Res..

[23] Shi PH (2016). Differential effects of temperature and duration of heat stress during anthesis and grain filling stages in rice. Environ. Exp. Bot..

[24] Krishana R, Ramakrishnan B, Reddy KR, Reddy VR (2011). High-temperature effects on rice growth, yield, and grain quality. Adv. Agron..

[25] Jagadish SVK, Murty MVR, Quick WP (2015). Rice responses to rising temperatures–challenges, perspectives and future directions. Plant Cell Environ..

[26] Tashiro T, Wardlaw I (1991). The effect of high temperature on the accumulation of dry matter, carbon and nitrogen in the kernel of rice. Aust. J. Plant Physiol..

[27] Kim K (1983). Studies on the effect of temperature during the reduction division and the grain filling stage in rice plants. II. Effect of air temperature at grain filling stage in indica-japonica crosses. Korean J. Crop Sci..

[28] Ito S (2009). Carbon and nitrogen transport during grain filling in rice under high-temperature conditions. J. Agron. Crop Sci..

[29] Chen JL (2017). Effects of short-term high temperature on grain quality and starch granules of rice (*Oryza sativa* L.) at post-anthesis stage. Protoplasma.

[30] Martinez-Sanz M, Gidley MJ, Gibert EP (2015). Application of small-angle X-ray and neutron scattering techniques to the characterization of starch structure: A review. Carbohydr. Polym..

[31] Yang XY (2016). Amylopectin chain length distribution in grains of japonica rice as affected by nitrogen fertilizer and genotype. J. Cereal Sci..

[32] Tang S (2019). Nitrogen fertilizer at heading stage effectively compensates for the deterioration of rice quality by affecting the starch-related properties under elevated temperatures. Food Chem..

[33] Zhang, X. A preliminary study on the effects of seed starch, protein, fat content on seed vigor of hybrid rice. Zhengjiang: Zhejiang A&F University (in Chinese) (2014).

[34] Chun A, Lee HJ, Hamaker BR, Janaswamy S (2015). Effects of ripening temperature on starch structure and gelatinization, pasting, and cooking properties in rice (*Oryza sativa* L.). J. Agr. Food chem..

[35] Yang JC, Zhang JH, Wang ZW, Zhu QS, Wang W (2001). Remobilization of carbon reserves in response of water deficit during grain filling of rice. Field Crop. Res..

[36] Tomlinson K, Denyer K (2003). Starch synthesis in cereal grains. Adv. Bot. Res..

[37] Zhang YB (2009). Yield potential and radiation use efficiency of “super” hybrid rice grown under subtropical conditions. Field Crop. Res..

[38] Wang XM, Zheng HB, Tang QY (2018). Early harvesting improves seed vigour of hybrid rice seeds. Sci. Rep..

[39] ISTA. *International Rules for Seed Testing*, International Seed Testing Association, Bassersdorf, Switzerland (2005).

[40] Hu J, Zhu ZY, Song WJ, Wang JC, Hu WM (2005). Effects of sand priming on germination and field performance in direct-sown rice (*Oryza sativa* L.). Seed Sci. Technol..

[41] Wei CX (2010). C-type starch from high-amylose rice resistant starch granules modified by antisense RNA inhibition of starch branching enzyme. J. Agric. Food Chem..

[42] Richards FJ (1959). A flexible growth function for empirical use. J. Exp. Bot..

[43] He, Z. F. & Zhang, D. Q. *The Chemistry and Detection Technology of Health Food*. China Light Industry Press, Beijing, 62–63 (1998).

[44] Juliano B (1971). A simplified assay for milled-rice amylose. Cereal Sci. Today.

[45] Raja RB (2017). Validation and applicability of single kernel-based cut grain dip method for amylose determination in rice. Food Anal. Method..

